# Analytical and Early Detection System of Infectious Diseases and Animal Health Status in Kuwait

**DOI:** 10.3389/fvets.2021.676661

**Published:** 2021-07-29

**Authors:** Ali Al-Hemoud, Manar AlSaraf, Mariam Malak, Musab Al-Shatti, Meshael Al-Jarba, Ahmad Othman, Hanadi Al-Shammari, Alya Al-Shatti

**Affiliations:** ^1^Environment and Life Sciences Research Center, Kuwait Institute for Scientific Research, Kuwait City, Kuwait; ^2^Systems and Software Development, Science and Technology Division, Kuwait Institute for Scientific Research, Kuwait City, Kuwait

**Keywords:** animal health, Kuwait, highly pathogenic avian influenza, foot and mouth disease, lumpy skin disease, glanders, MERS-CoV

## Abstract

This study aimed at the development of an analytic web-based system for the assessment of animal health in Kuwait. The data sources were based on the World Organization for Animal Health (OIE) and the World Animal Health Information System (WAHIS) repository with data gathered for the period (2005–2020). An on-line web-based system using TABLEAU Creator was developed for monitoring and surveillance of animal disease outbreaks. Five animal diseases were identified in Kuwait; namely, HPAI, FMD, glanders, LSD and MERS-CoV. The highest numbers of outbreaks were recorded for HPAI, followed by FMD. Examples of spatio-temporal visualizations of the web based mappings are presented and include disease cases, number of outbreaks and farm locations, among other features. The web-based system can serve as a monitoring tool to easily display the status of animal health in Kuwait. It can also serve to quickly identify and track disease outbreaks and monitor the spread patterns of new or emerging animal diseases between neighboring countries.

## Introduction

Ecological health is a fundamental asset for the sustainability of life for all species on Planet Earth. Within this respect, animal health is an integral component of ecological health. In particular, for a very small country such as Kuwait (17,818 km^2^) which is surrounded by much larger countries such as Saudi Arabia, Iraq and Iran, makes animal health a national asset and ranks high in importance in the same class of local natural resources such as oil. For instance, both Kuwait and Saudi Arabia experienced several highly pathogenic avian influenza (HPAI) H5N1 outbreaks in poultry and falconry sectors (1, 2). The most recent foot and mouth disease (FMD) cases were identified in cattle in Saudi Arabia (3–5) and Iraq (6, 7) in 2016. Middle East respiratory syndrome coronavirus (MERS-CoV) was first identified in Saudi Arabia in September 2012 (8). The risk of returning Hajj pilgrims contacting MERS-CoV in Iran was high (9). With the propagation of emerging diseases in the Middle East (e.g., MERS-CoV) in recent years, it became quite clear that there is an urgent need to understand and monitor the status of animal health from historical perspective and to build an early detection system (EDS) for animal disease outbreaks, with the collective goal to track the status of animal health in Kuwait. This endeavor was embarked upon by researchers from the Kuwait Institute for Scientific Research (KISR). A literature review revealed two classes of analytic-based systems undertaken by (a) global organizations via integrated efforts, and (b) regional agencies/individual researchers via local efforts. The Global Early Warning System (GLEWS) is an example of global information system which is a joint effort between food and agriculture organization (FAO), world organization for animal health (OIE), and world health organization (WHO) initiative for transboundary animal diseases (10), bringing together human/veterinary public health systems to share zoonotic disease outbreak information/epidemiological and risk analysis. Its aim was to deliver early warning messages to the international community at risk of transboundary animal diseases. The Emergency Prevention System (EMPRES) for Transboundary Animal and Plant Pests is another earlier example of a global effort by the FAO organization, with emphasis on the prevention of emergencies due to transboundary epidemic diseases and/or food security (11, 12). The Predictive Livestock Early Warning System (PLEWS) is an example of a regional effort by researchers in Kenya for monitoring of forage condition and early warning system for the reduction of livestock loss and improvement of their resilience (13). In the Middle East, the majority of countries do not have an on-line monitoring system for animal disease outbreaks and only few countries in the region regularly use geographic information systems in their routine surveillance (14). Kuwait's Public Authority of Agriculture Affairs and Fish Resources (PAAF) which is the official local authority for animal health surveillance and veterinary services rely on a passive surveillance system for reporting disease occurrences provided by farmers and veterinarians. An active monitoring system is required to better understand the spreading pattern of a disease and increase the speed of response in the case of a disease emergency. A good monitoring system should provide fast tracking of disease outbreaks and assist decision makers in understanding and explaining disease dynamics and spreading patterns.

Recent reports have called upon the need to increase investment in early warning and detection systems of animal health, with the goal to provide information enabling action to be taken at the national, regional and global levels (15). Furthermore, advanced disease intelligence systems have been also advocated for emerging infectious diseases, as they are among the most damaging and costly natural forces (16). Bisson et al. (17) reported that, over the last 60 years, more than half of the emerging infectious diseases appearing in humans have been transmitted from animals, of which 72% are of wildlife origin. The One Health concept recognizes that the health of people is connected to the health of animals and the shared environment (18). It is a collaborative and interdisciplinary approach with the goal of achieving optimal health outcomes by recognizing the interconnection between the environment, people, animals, and plants (19–21). A national disease registry of zoonotic diseases must be developed and the central challenge in the rapid detection of emerging zoonoses is to create a robust surveillance network (22).

In light of the aforementioned, the objective of this study is to develop an on-line web based system to ease the early detection of animal diseases in Kuwait. The on-line monitoring system can serve as the base for early detection and identification of animal diseases in Kuwait. At this early stage in the development of the system the intention is to apply a dynamic “early detection” system that can geographically monitor the spread of disease outbreaks.

## Methods

### Database Reporting System

The epidemiologic database reporting system in this study followed the methodology framework established by OIE and utilized the data stored in the OIE WAHIS system for the state of Kuwait (23–25). Reported outbreaks were collected for 16 years (2005–2020). Based on the OIE repository data from 2005 to 2020, five diseases were registered in Kuwait, namely, HPAI, FMD, glanders, MERS-CoV, and lumpy skin disease (LSD). Information was organized around the release of any given outbreak in a given year, with the following attributes: region of outbreak occurrence, starting date of outbreak, outbreak status, epidemiological unit, affected animals, and description of affected animal population. In this respect, an epidemiological unit is intended to be a group of animals with a defined epidemiological relationship that share approximately the same likelihood of exposure to a pathogenic agent. The affected animals consist of species' type and defining epidemiologic characteristics such as cases (an individual animal infected by a pathogenic agent with and without clinical signs), deaths (due to disease causes out of confirmed cases), susceptible [population at risk or number of animals from susceptible species in on-going active outbreak(s) during the reporting period], euthanized and disposed of (any procedure causing the death of an animal, and based on human decisions as related to the susceptible animal population); in addition, outbreak statistics included morbidity rate (i.e., cases/susceptible population).

### Web-Based Analytic System

The analytic epidemiologic system was implemented in an online TABLEAU (26) environment. In essence, TABLEAU is the most versatile web-based data visualization tool which interfaces with the epidemiologic database established for this project. We have acquired a TABLEAU Creator License which includes a TABLEAU desktop, online server and TABLEAU viewer. The analytic system is organized around two parts, namely, summary statistics and outbreak statistics. The “dimensions” that we included in Tableau were: farm name, farm location, coordinates (latitude, longitude) and date. The “measures” that we used in the statistics include animal disease, number of outbreaks, number of susceptible animals, species, number of vaccinations and route of administration. The data source was acquired from OIE focal point in Kuwait (PAAF). Data were compiled from immediate notification (within 24 h), 6-month, and annual reports. The diseases recorded in TABEAU included OIE-listed diseases (first occurrence of the disease in the country, reoccurrence after a period of absence, sudden or unexpected change in the distribution of the disease) and emerging animal diseases. Although our current analysis is based on historical reported outbreaks (2005–2020) of notifiable diseases, the system can accommodate immediate disease occurrences; an important management tool to find patterns and track the spread of disease for early monitoring and surveillance.

The summary statistics consist of a tabular display of the total count of animal cases, deaths, euthanized and destroyed. The disease dashboard is a graphical display (bar format) with filter options where the x axis is designated for the years of outbreak from 2005 to 2020, and the statistics, namely, sum cases, sum susceptible, sum deaths and sum euthanized, for every disease is displayed on the y axis. The primary filter is the disease with an all option across all diseases.

The outbreak statistics dashboard is a graphical display of the regional map in Kuwait where the outbreak has taken place. The primary filter is the disease in question. The secondary filter is used for the year of outbreak and a third filter gives the exact location of the outbreak. The circle size corresponds to the number of cases in a particular geographical region of an outbreak. This can be done for any of the statistics required (sum confirmed cases, sum susceptible, sum deaths or sum euthanized) and upon clicking the circle a window opens with a display of all the disease records occurring at that particular point in time including the count of susceptible cases, sum cases, deaths and destroyed. In addition, the location and locality name is also shown. The occurrence of a particular geographical area in a given year for a given disease will provide a display of all measures within the region during the specified year.

## Results

### History of Outbreak, Disease and Affected Geographical Area Information

Based on the observation date, HPAI and FMD are responsible for the most outbreak counts (Table 1). The outbreak history for HPAI was: 1 in 2005, 18 in 2007, 1 in 2016, 1 in 2019 and 1 2020. FMD had 1 outbreak in 2009, 1 in 2011, 2 in 2012 and 5 in 2016. Five outbreaks of the emerging MERS-CoV occurred between 2013 and 2015. LSD occurred in 2014 and 2015 resulting in 4 outbreaks. Finally, glanders had 1 outbreak that occurred in 2019. As such, the animal species infected in Kuwait were birds (due to HPAI), cattle (due to FMD and LSD), camels (due to MERS-CoV), and horses (due to glanders).

**Table 1:** Animal disease outbreaks and locations in Kuwait (2005–2020).

	**No. outbreaks**	**Location**
HPAI	22	All 6 governorates^*^
LSD	4	Jahra
MERS-CoV	5	Capital, Jahra, Ahmadi
FMD	9	Jahra
Glanders	1	Ahmadi

* *The six governorates in Kuwait are: Capital, Jahra, Ahmadi, Farwaniya, Mubarak Al-Kabeer, Hawalli*.

Additionally, HPAI was observed in all of the 6 governorates in Kuwait. FMD and LSD were concentrated in Jahra governorate only, while MERS-CoV was observed in three governorates, namely, Capital, Jahra and Ahmadi. Finally, the one outbreak of glanders occurred in Ahmadi governorate only. It should be noted that Jahra is the largest governorate in Kuwait and occupies the northern territory, and Ahmadi fills the southern region. Farwaniya and Capital governorates are located in the middle between Jahra and Ahmadi.

### Disease Measure Statistics

#### Distribution of Outbreaks

**Figure 1:**
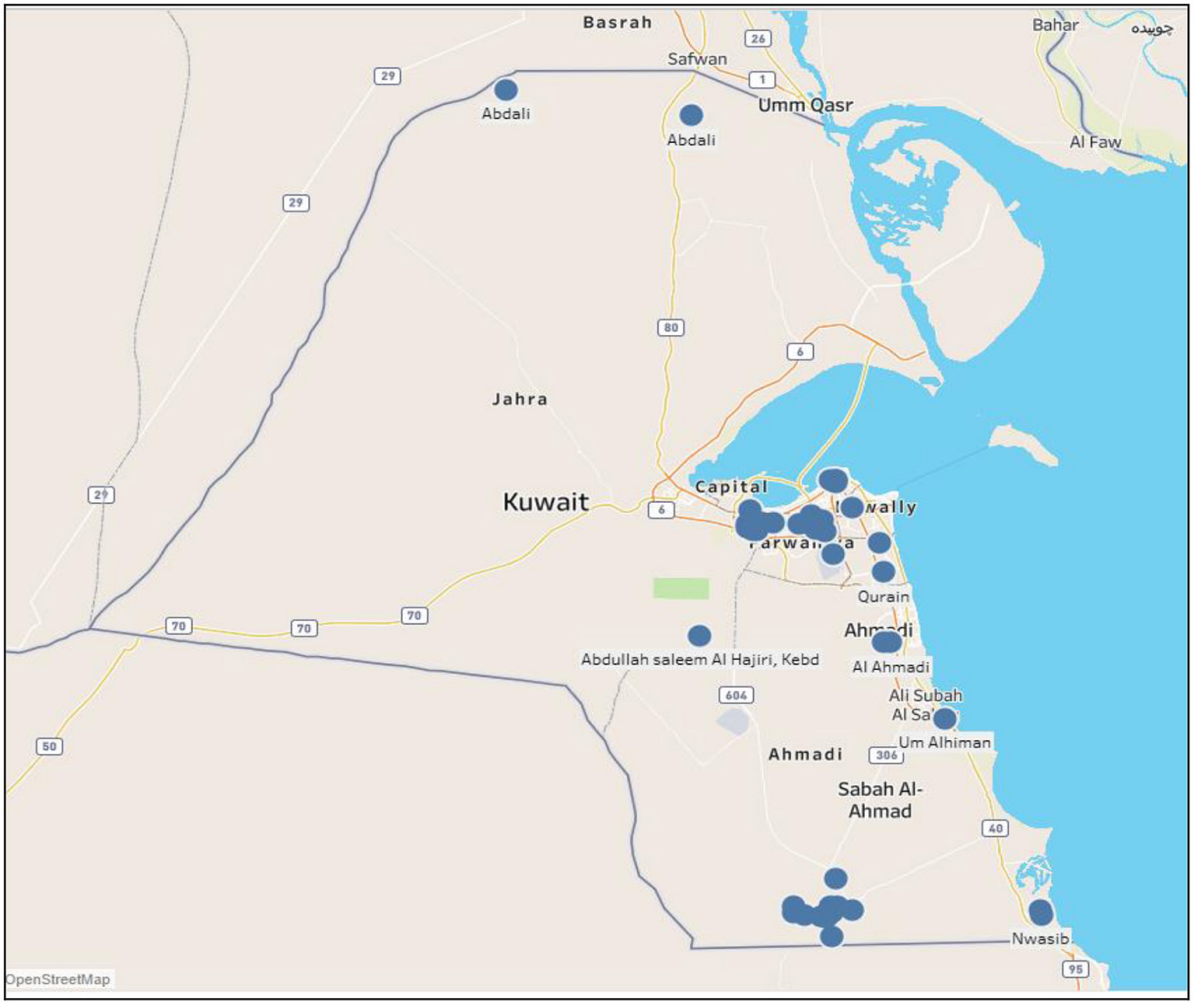
Spatial distribution of all animal diseases in Kuwait (2005–2020). Circles indicate the location of farms with outbreaks.

Figure 1 shows the geographical locations of outbreaks of all five animal diseases; namely, foot and mouth, HPAI, MERS-CoV, lumpy skin and glanders in Kuwait during the period 2005 to 2020. As shown, most outbreaks occurred in Ahmadi governorate (south of Kuwait-Wafra area) and Farwarniya governorate (i.e., Sulaibiya), but due to the difference in area, Sulaibiya is considered denser with outbreaks. In addition, HPAI hit Farwaniya governorate in 2007. Also, the Capital governorate (i.e., Kuwait City) registered some disperse outbreaks that occurred in different years. Other outbreaks occurred at the borders specifically North of Kuwait in Abdali area whereas other outbreaks occurred at the Kuwaiti-Saudi border namely at Nwasib. The rest of the outbreaks occurred at random locations all over Kuwait.

**Figure 2:**
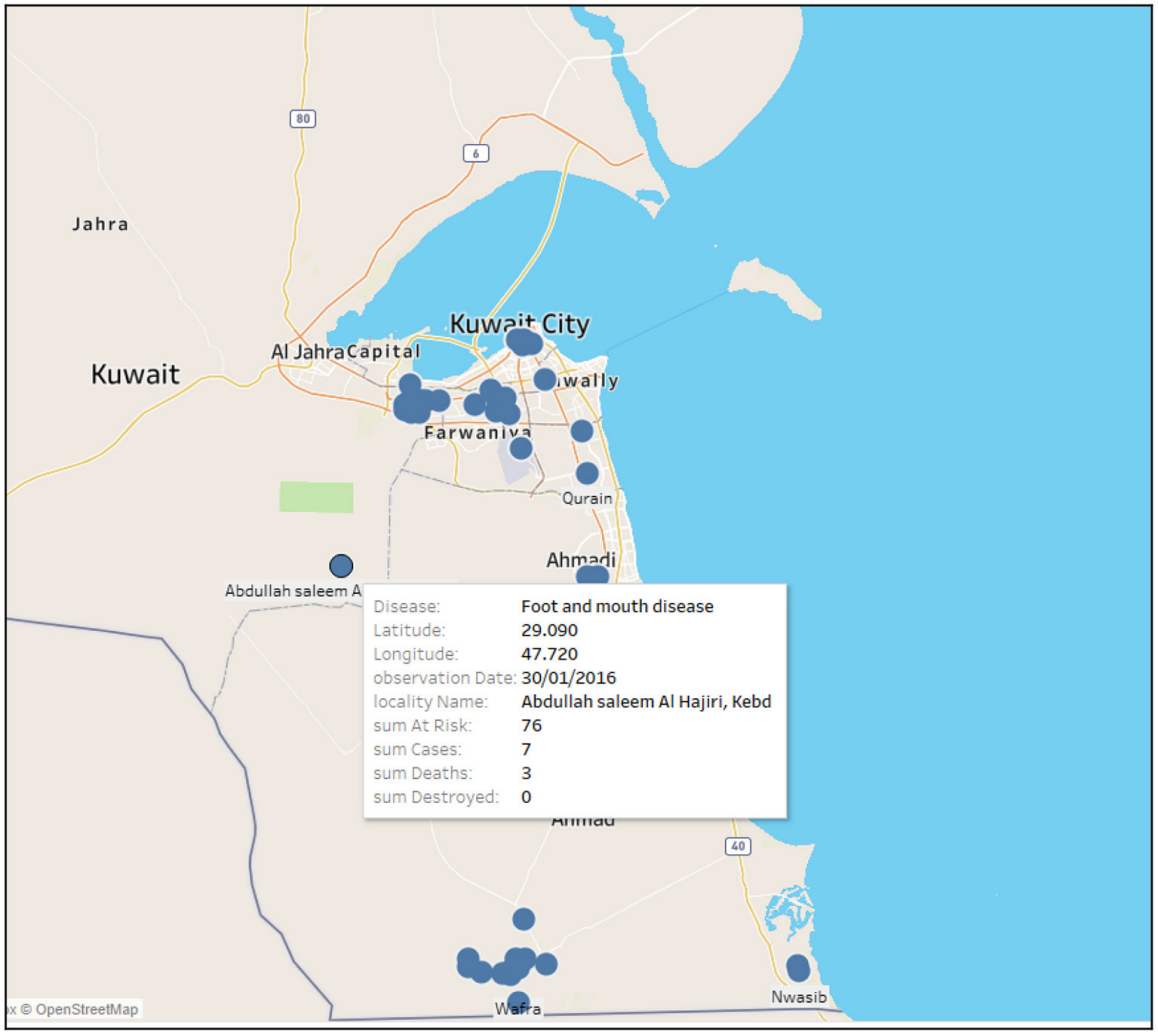
Example of a web based analytic system map showing the coordinates of FMD outbreak at a specific farm on 30/01/2016 with all outbreak information [i.e., sum at risk (susceptible), confirmed cases, deaths, destroyed].

Figure 2 shows an example of the output of the web based analytic system that relates different information of a disease (i.e., FMD) into a spatial context. For instance, FMD outbreak occurred in Abdullah saleem Al Hajri, Kebd farm on the 30th of January 2016. It consisted of 76 risk cases in which seven were confirmed; three out of the seven died and none were destroyed. The size of the circle represents the total number of outbreaks at a specific farm (i.e., the larger the circle the greater the number of outbreaks).

#### Data Visualization of HPAI

**Figure 3:**
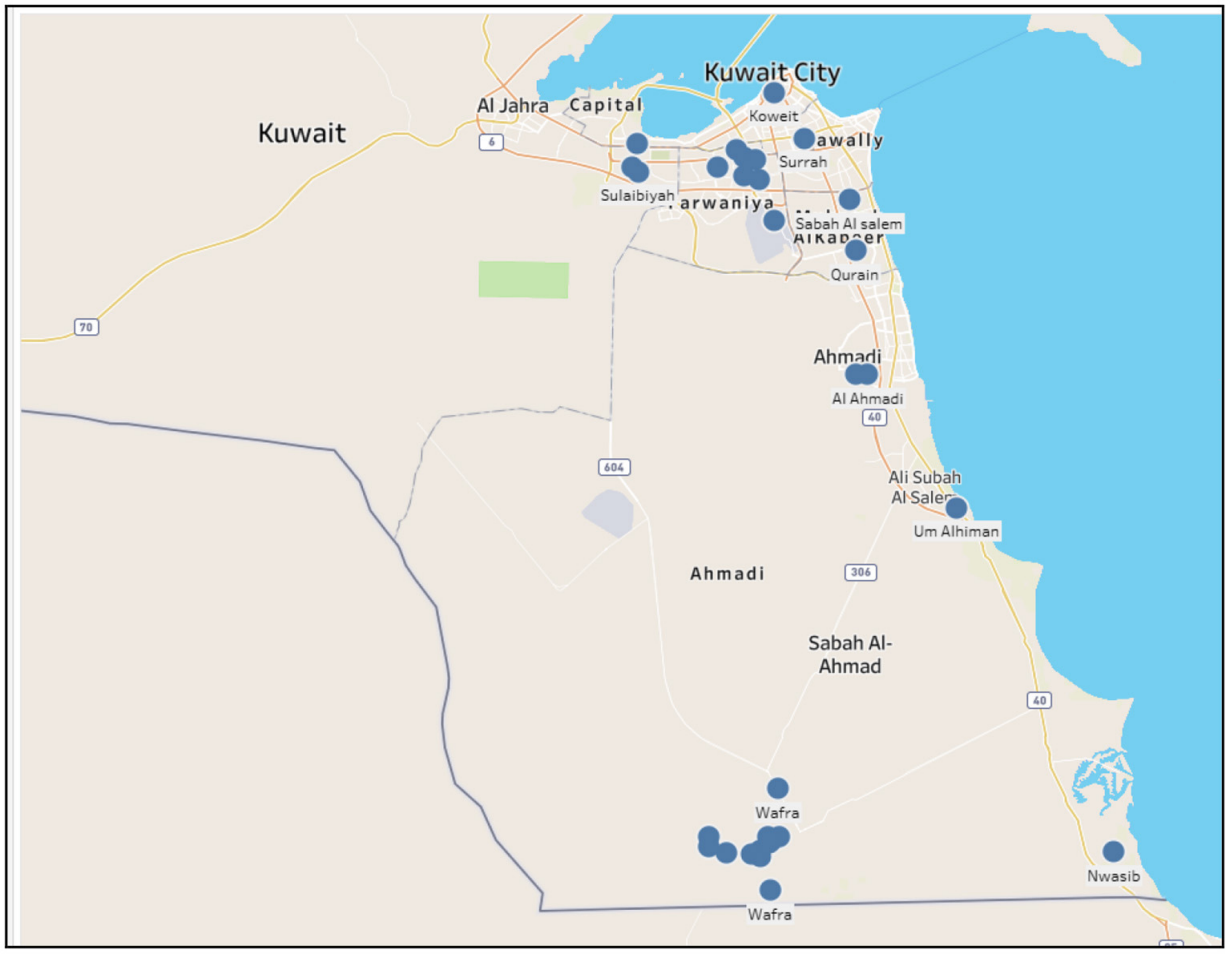
Highly pathogenic avian influenza outbreaks in Kuwait during the period 2005–2020.

The first outbreak of HPAI occurred in 2005 in two different locations with only 2 cases. The second outbreak of the HPAI occurred in 2007 especially in Wafra where most farms are located with a total number of 135 cases and the sum at risk (total number of susceptible animals in a single farm) reached almost 470,000. Total deaths were 112 and almost 467,000 were destroyed. The third outbreak occurred in 2016 with 200 cases. An outbreak of HPAI also occurred in 2019. Although the sum at risk reached up to 14,000, only eight cases were registered and the rest have been destroyed. A final outbreak occurred in 2020 with 112 cases at risk in which eight died and the rest were destroyed. Table 2 shows the summary statistics of all 5 animal diseases in Kuwait and Figure 3 shows the map of HPAI outbreaks.

**Table 2:** Summary statistics of all animal diseases in Kuwait (2005–2020).

	**Sum at risk (susceptible)**	**Sum confirmed**	**Sum deaths**	**Sum destroyed**
HPAI	483,557	369	272	481,297
LSD	6,642	415	27	98
MERS-CoV	63	5	0	0
FMD	8,161	771	44	0
Glanders	44	6	0	6

#### Data Visualization of Lumpy Skin Disease

All lumpy skin disease occurred in Sulaibiya area in which the first outbreak occurred in 2014. Although there were 342 risk cases, only 75 cases were registered in which seven died and 68 were destroyed. The other three outbreaks occurred in February 2015 with a total of 340 cases.

#### Data Visualization of MERS-CoV

**Figure 4:**
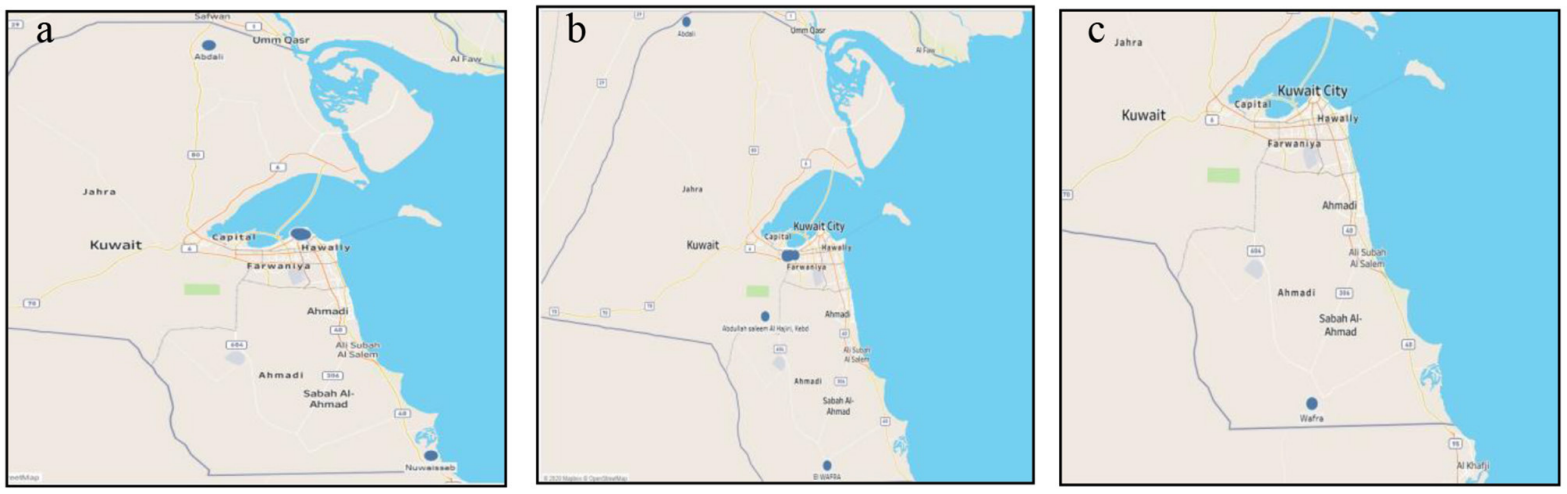
Middle East respiratory syndrome coronavirus outbreaks **(a)**; Foot and mouth disease outbreaks **(b)**; Glanders outbreaks in Kuwait during the period 2005–2020 **(c)**.

Most MERS-CoV outbreaks occurred in the Capital governorate (Kuwait City) from the years 2013–2015. However, the actual number of cases was not registered. One outbreak occurred on 5/6/2014 in two different locations with 63 susceptible cases and only five actual cases; none of which died nor have been destroyed. Figure 4a shows the map of MERS-CoV outbreaks.

#### Data Visualization of Foot and Mouth Disease

One FMD outbreak occurred in 2009 in Sulaibiya (Farwaniya governorate) with more than 2000 susceptible cases but only 60 were registered. Table 1 shows the statistics of the registered outbreaks. In fact, most outbreaks occurred in Sulaibiya area especially in January 2016 with a total of 711 cases and 6,101 cases at risk. One outbreak occurred in Sulaibiya in 2011. Two other outbreaks occurred in 2012 on both borders (Kuwait-Iraq and Kuwait-Saudi); however, the actual number of risk cases is unknown. Figure 4b shows the outbreaks of FMD that occurred in the North, South and center of Kuwait.

#### Data Visualization of Glanders Disease

There is only one glander disease outbreak that occurred recently in 2019 in Wafra area. Only 44 cases were at risk and six were confirmed. All six cases have been destroyed. Figure 4c shows the outbreak of glanders disease.

#### Susceptible Cases

**Figure 5:**
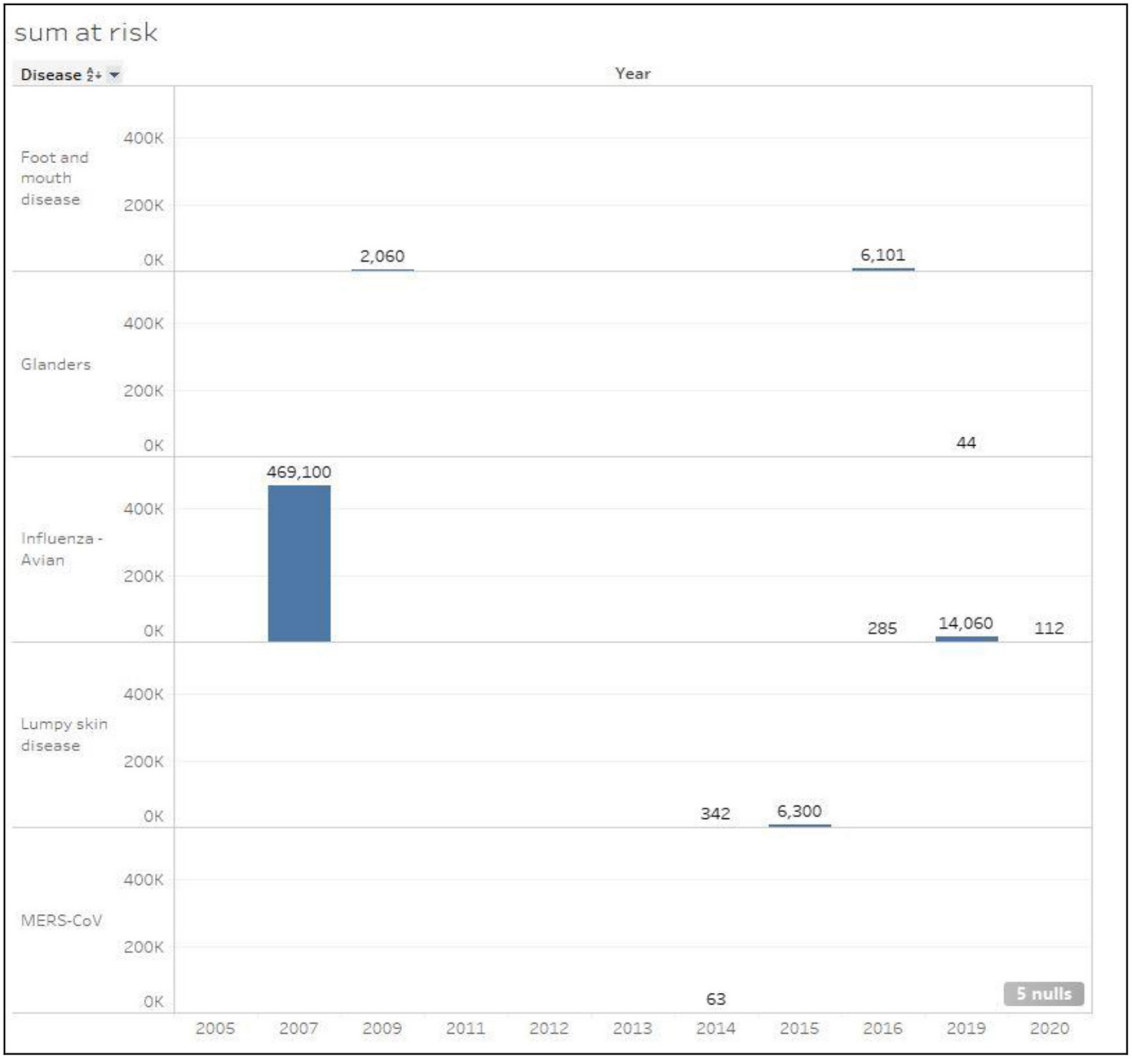
Total number of susceptible cases classified by disease in Kuwait during the period 2005–2020.

Figure 5 shows the total number of susceptible cases classified by disease type for the period (2005–2020). Clearly, HPAI disease in 2007 included by far the most susceptible cases with a total of 469,100, followed by the same disease in 2019 with a total of 14,060 cases. Foot and mouth disease is considered the second highest disease with a total of 8,161 susceptible cases. However, in 2015, lumpy skin disease scored the third highest number of susceptible cases (6,300). MERS-CoV and Glanders diseases had the least number of susceptible cases with 63 and 44 cases consecutively.

#### Infected and Euthanized Cases

**Figure 6:**
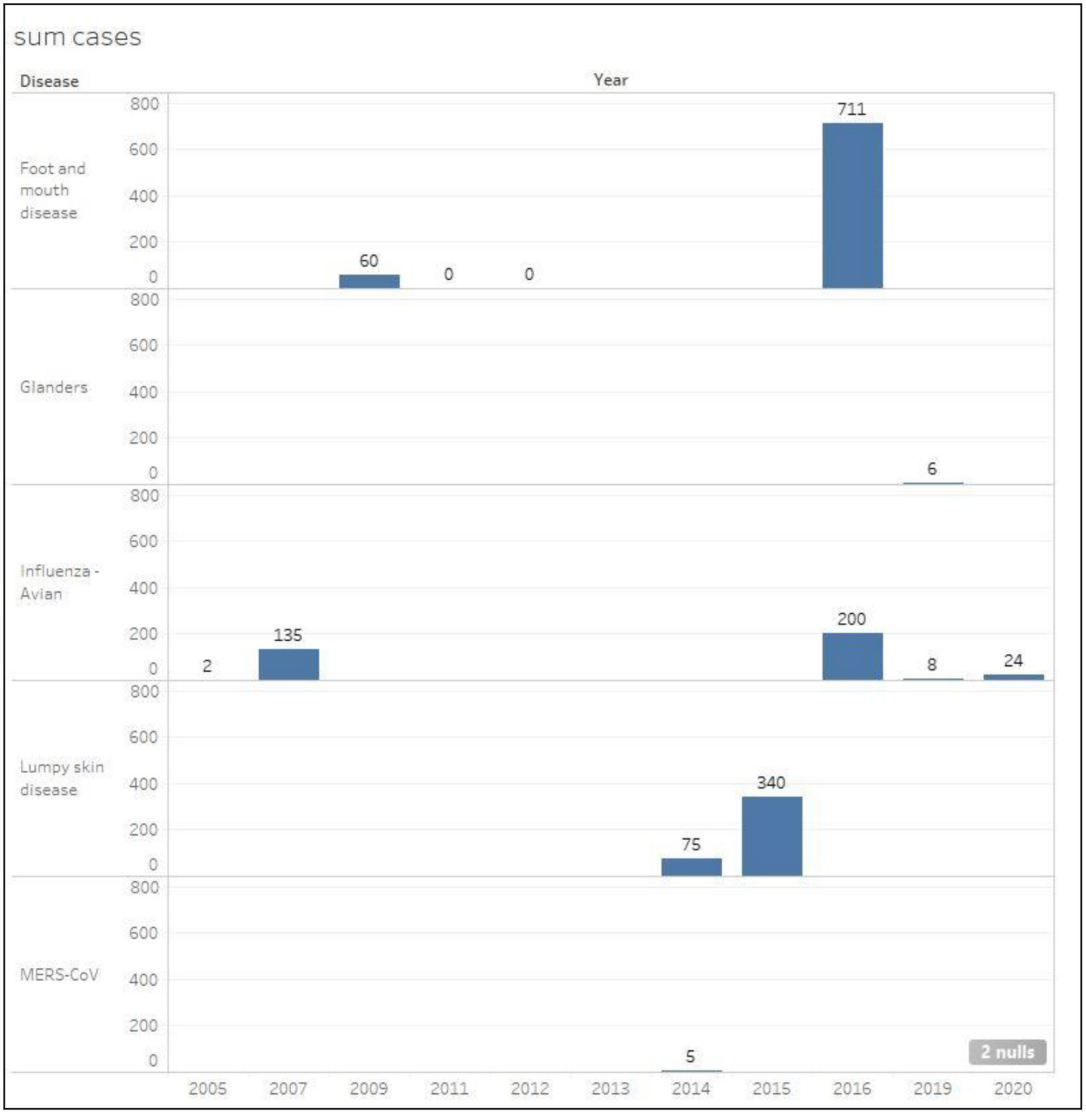
Total number of infected cases classified by disease in Kuwait during the period 2005–2020.

**Figure 7:**
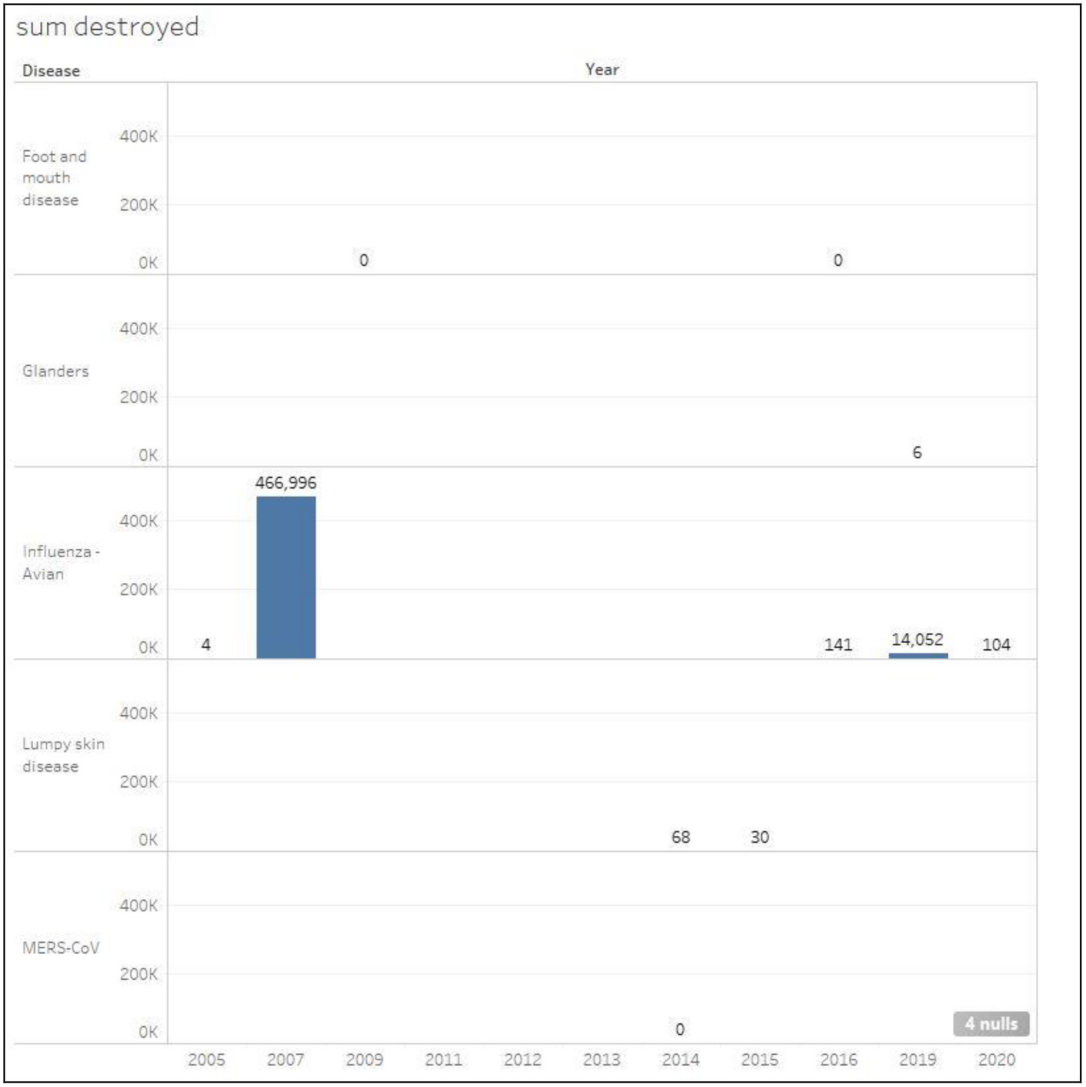
Total number of euthanized cases classified by disease in Kuwait during the period 2005–2020.

Figures 6, 7 show the total number of infected and euthanized cases for all diseases during the period (2005–2020). Foot and mouth disease scored the highest number of infected cases with a total of 711 in 2016. Lumpy skin disease scored the second highest in 2015 with a total of 340 cases. The highest numbers of euthanized cases were by far registered for HPAI during 2007 (466,966) and 2019 (14,052).

## Discussion

The developed early detection system can serve as surveillance and monitoring tool to determine the status of local animal health to quickly identify disease outbreaks and monitor the spread patterns. Although the developed on-line system was based on passive surveillance from historical notifiable OIE diseases for the period (2005–2020), it can also accommodate active surveillance modalities. Immediate notifications (within 24 h) from farm owners of the first incursion of a particular disease can be immediately recorded in the system at the earliest possible time to provide a quick overview of the early detection of changes in animal health in a given area. For instance, clinical investigations at the Friday bird market by PAAF officials on 24 January 2019 identified a captive pet bird with HPAI and 8 dead birds. Officials quickly took stringent control measures and euthanized all birds on the same day (14,052 birds); thus, controlling the spread of infection to major local poultry farms located in central, north and south Kuwait. The early detection system supports the visualization of disease outbreaks to allow the constant update of the disease situation, and interventions should animal health threats occur. The system can also provide useful information for advanced methods that support animal health surveillance, such as cluster analysis of epidemiological events, regression analysis and risk assessment. The idea of establishing an early warning center between the Gulf Cooperation Council (GCC) countries was proposed by Kuwait. The “Gulf Early Warning Center for Transboundary Animal Diseases” was established in 2012 under the responsibility of the deputy director general for livestock at PAAF. To the best of our knowledge and through our discussions with PAAF crisis and emergency response department no web-based integrated system exists between neighboring countries. Our developed system can incorporate animal health data from neighboring countries and can serve as the base for transboundary animal disease communication.

OIE encourages member countries to build their own national animal health information system and not depend solely on WAHIS (23). The developed early detection system can be utilized for early warning as a secured online notification system between farmers, veterinary services, and the country's official focal point. For instance, the system can provide alert messages for exceptional epidemiological events, first occurrence or re-occurrence of animal diseases or infections, and sudden and unexpected increase in animal morbidities or mortalities. The system can be very helpful to disseminate information between neighboring GCC countries related to animal species affected, geographical coordinates of the outbreaks, laboratory tests, and number of vaccinations. Quantitative data of diseases can be shared to meet the needs of national veterinary authorities to allow a better understanding of the epidemiological situation. Neighboring countries can also use standardized report forms in their native language to ensure consistency and homogeneity of shared information.

A historical analysis of diseases in Kuwait in the last 16 years revealed the emergence of five diseases, namely, FMD, highly pathogenic avian influenza, MERS-CoV, LSD and glanders. These diseases reflect the vulnerability of animal assets in Kuwait from various perspectives and the need for action for the protection of these assets.

With respect to FMD, Rweyemamu and Astudillo (27) reported that the presence of this disease in a particular country is a reflection of its economic structure. It is a severe, highly contagious viral disease of livestock caused by the aphthorivus organism of the family Picornaviridae that has a significant economic impact, affecting cattle, swine, sheep, goats and other cloven-hoofed ruminants (28). The morbidity rate may approach 100% in susceptible cattle populations. The goal of Kuwait should be to eradicate the roots of this disease given the economic profile of the country in terms of GDP per capita, its non-agriculture-based economy and the potential economic losses of FMD. Therefore, there should be a call for action to improve the public health surveillance and control measures of such a threat in Kuwait to be in line with international recommendations (27, 29).

LSD is another infectious disease of cattle caused by a virus belonging to the genus Capripoxvirus and family Poxviride (30). It is usually transmitted via arthropods with direct and indirect transmission occurring. Although the roots of LSD are in the African desert, it is believed that infected insects such as Stomoxys calcitrans can cause the infection carried via natural forces such as wind (31). Although the morbidity rate of the LSD outbreaks in Kuwait is small (6.2%) relative to those reported by OIE (rates vary between 10 and 20%) (32) precautions should be put in place to protect the precious smaller number of animals in Kuwait.

Another perspective emanates from the fact that because Kuwait is a small country with limited animal assets and surrounded by much larger countries (e.g., Saudi Arabia), which makes the country very vulnerable to the quick spread of any emerging epidemics such as the MERS-CoV and highly pathogenic Avian influenza that greatly influences the status of animal health in Kuwait. As such, it is of paramount importance to track the status of animal health to act decisively at the right time. This is particularly important in the case of HPAI due to the great losses in the susceptible population because of the aforementioned reason and the added reason of migratory birds. For instance, a confirmation of H5N1 in a flamingo in November 2005 marked the first detection of the virus in the Arabian Gulf. The flamingo was found along the coastal area of Kuwait, a common visiting place for flamingoes mainly in the tidal mud flats. On the same day a falcon imported from an Asian country tested positive for H5N2 and was quarantined at the Kuwait airport. In February 2007 two captive falcons were confirmed with HPAI, one was identified at the Friday market and the other was at a local farm south of Kuwait. The Kuwait virus isolates were closely related to isolates from central Asia and were likely vectored by migratory birds (1). Studies report more than 300 bird species in Kuwait; most of them are migratory in nature (33, 34). Kuwait is located within two primary migratory bird pathways, the Black Sea/Mediterranean flyway and the East Africa—West Asia flyway. Some of the locations where migratory birds were identified include Abdali and Wafra, north and south of Kuwait, respectively, in close proximity to major poultry farms. Tidal mud flats and Sabkhas are a characteristic ecosystem of Kuwait and attract a large number of migratory birds throughout the year. There are extensive mudflats, especially in the northern coastal area. The breeding population of Crab plover (*dormas ardeola*) on Bubiyan island is probably the biggest in the world, while Kubbar island is historically home for colonies of breeding seabirds, where thousands of bridled tern (*Onychoprion anaethetus*), white-cheeked tern (*Sterna repressa*) and hundreds of lesser crested tern (*Thalasseus bengalensis*) breed annually. By far, HPAI recorded one of the highest numbers of outbreaks (22) between 2005 and 2020, with a very high loss rate amounting to 99.6% including euthanized and disposedcases. In total, Kuwait lost close to half million birds due to HPAI. Avian influenza results from infection by viruses belonging to the species influenza A virus, genus influenza virus A and family Orthomyxoviridae (35). In light of the above, it appears essential to combat the avian influenza to protect the domestic bird population from the deadly virus (36). Thus, one must build upon the experience of others who identified the gaps in knowledge which can assist in the design of optimal surveillance systems (37). In contrast to HPAI, MERS-CoV had five outbreaks with a morbidity rate of 7.9% due to the emergence of the disease in Saudi Arabia and occurring in 2014 (38). Also known as the camel flu, the disease is a viral respiratory infection caused by the MERS-betacoronavirus derived from bats. No cases have been reported in Kuwait in the last 6 years. In addition, FMD resulted in a mortality rate of 9.4% within its outbreaks.

Although Kuwait does not have an agriculture-based economy, an outbreak of glanders appeared for the first time in 2019 with six reported cases among a susceptible population of 44 resulting into a morbidity rate of 13.6%, a considerably high rate. Glanders is caused by Burkholderia mallei, an infectious and contagious disease of equines (39). Thus, one is indeed in need to be in surveillance of such diseases particularly when they appear for the first time.

The web-based analytic system developed in this study is paramount to the continuous monitoring and implementation of control measures for the protection of animal welfare. In particular, the EDS dashboard will be a key to track key diseases regionally and globally. For example, glanders has appeared only recently for the first time in 2019. Since this disease originates in agriculture-based economies utilizing horses, donkeys and mules, this disease is not typical to be present in arid regions like Kuwait as it is not an agriculture-based economy and relies upon imports to fulfill its food requirements. Furthermore, the horses impacted in Kuwait are Arabian horses and are of expensive breed hence highlighting the economic impact of the disease. Therefore, upon investigation of the source of the bacteria it was found that Turkey had several outbreaks in 2019 particularly in the second half of the year. Furthermore, Iran had cases of glanders in 2019, particularly in the first half of the year. Therefore, it is crucial to install a stringent public health surveillance system to avoid the spread of such animal diseases.

In light of the above, it appears that the animal diseases observed in Kuwait are impacted by the animal disease activity in the region surrounding Kuwait as well as the migrating birds passing through Kuwait. Therefore, the EDS developed in the current study should be extended in a dynamic fashion to the disease activities appearing in the region surrounding Kuwait. Future research will address these issues and will attempt to capitalize upon the extra-sensory capabilities of animals for the protection of Kuwait against infectious diseases and national emergencies.

## Conclusion

This study presents the development of an “early detection” system aimed at monitoring animal disease outbreaks in Kuwait. Reported outbreaks were collected for 16 years (2005–2020) from OIE which was submitted by the Kuwait focal point at PAAF. TABLEAU Creator was used to visualize disease spreading patterns across various governorates and farms in Kuwait. Five animal diseases were identified in Kuwait; namely, HPAI, FMD, glanders, LSD and MERS-CoV. The on-line system is considered an important tool to visual historical trends of animal health and to also track the spread of diseases at early stages to effectively identify animal threats and develop proper control measures. For future purposes, the system can be used as an “early warning” tool for the prediction and alert of new or emerging diseases.

## Data Availability Statement

The original contributions presented in the study are included in the article/supplementary material, further inquiries can be directed to the corresponding author/s.

## Author Contributions

AA-H wrote the manuscript. MA entered and analyzed the collected data in TABLEAU. MM, MA-J, HA-S, and AA-S assisted in data collection. MA-S and AO used GIS to manipulate and display the data. All authors contributed to the article and approved the submitted version.

## Conflict of Interest

The authors declare that the research was conducted in the absence of any commercial or financial relationships that could be construed as a potential conflict of interest.

## Publisher's Note

All claims expressed in this article are solely those of the authors and do not necessarily represent those of their affiliated organizations, or those of the publisher, the editors and the reviewers. Any product that may be evaluated in this article, or claim that may be made by its manufacturer, is not guaranteed or endorsed by the publisher.
